# Stress hyperglycemia ratio predicts mid- to long-term mortality in first-hospitalized type 2 diabetes: Nonlinear threshold and prognostic value

**DOI:** 10.1371/journal.pone.0351307

**Published:** 2026-06-30

**Authors:** Xiaohan Li, Radhakrishnan Muthukumar, Isaraporn Thepwongsa

**Affiliations:** 1 Department of Community, Family and Occupational Medicine, Faculty of Medicine, Khon Kaen University, Khon Kaen, Thailand; 2 Academic Affairs, Faculty of Medicine, Khon Kaen University, Khon Kaen, Thailand; University of Diyala College of Medicine, IRAQ

## Abstract

**Background:**

The stress hyperglycemia ratio (SHR) can more accurately reflect acute glycemic dysregulation by incorporating chronic glycemic levels. However, its association with mid- to long-term all-cause mortality in first-hospitalized patients with type 2 diabetes mellitus (T2DM), as well as its nonlinear characteristics, threshold effect and incremental predictive value, remain unclear.

**Methods:**

This single-center retrospective cohort study enrolled 1,147 first-hospitalized T2DM patients. The prognostic value of SHR was evaluated using restricted cubic spline analysis, threshold effect analysis, Cox regression, subgroup analysis, sensitivity analysis and time-dependent receiver operating characteristic (ROC) curves.

**Results:**

SHR was nonlinearly associated with all-cause mortality at all follow-up time points, with an optimal threshold of 1.08 (both P < 0.05). Each 0.1-unit increase in SHR was associated with a 10% higher risk of 3-year and 5-year mortality (HR = 1.10). SHR ≥ 1.08 was associated with increased risks of 3-year and 5-year mortality (HR = 2.38 and 2.28, both P < 0.001). Sensitivity analysis confirmed the robustness of results. Subgroup analysis showed that myocardial infarction, congestive heart failure and cerebrovascular disease significantly modified the prognostic effect of SHR. Time-dependent ROC demonstrated favorable predictive performance with AUC > 0.80 at all time points. Moreover, SHR resulted in modest improvements in AUC (0.825 → 0.835; 0.813 → 0.823) and C-index (0.792 → 0.802), outperforming traditional glycemic indicators.

**Conclusion:**

SHR is independently and nonlinearly associated with mid- to long-term all-cause mortality in first-hospitalized T2DM patients (threshold = 1.08), and may provide incremental prognostic information for risk stratification.

## Introduction

Type 2 diabetes mellitus (T2DM) represents a major global public health challenge, with a rapidly increasing prevalence over recent decades. According to the International Diabetes Federation, approximately 537 million adults were living with diabetes worldwide in 2021, and this number is projected to rise to 783 million by 2045, of whom 90–95% are diagnosed with T2DM [1–5]. This escalating epidemic has imposed substantial healthcare costs and long-term socioeconomic burdens worldwide.

The adverse prognosis of T2DM is largely attributable to chronic hyperglycemia and its associated metabolic disturbances, including insulin resistance, dyslipidemia, and persistent low-grade inflammation. Sustained metabolic imbalance promotes endothelial dysfunction, oxidative stress, and the accumulation of advanced glycation end products, thereby accelerating the development of both microvascular and macrovascular complications. Among these, cardiovascular disease remains the leading cause of morbidity and mortality in patients with T2DM [6–9]. Importantly, the risk of adverse outcomes becomes particularly pronounced during acute illness requiring hospitalization, when underlying metabolic vulnerability may be further exacerbated.

During hospitalization, patients with T2DM frequently experience significant glycemic fluctuations triggered by acute medical conditions, comorbidities, and physiological stress responses. Stress hyperglycemia, characterized by transient elevation of blood glucose in response to acute stress, results from increased insulin resistance and activation of counter-regulatory hormones such as cortisol and catecholamines. These stress-related metabolic alterations aggravate cardiometabolic instability and are strongly associated with unfavorable clinical outcomes [10–15]. Therefore, accurate evaluation of stress-related glycemic dysregulation during hospitalization is crucial for early risk stratification and long-term prognostic assessment.

However, traditional glycemic indicators have inherent limitations in this setting. Admission blood glucose (ABG) reflects acute glycemic status but is highly susceptible to transient stress-related influences, whereas glycated hemoglobin (HbA1c) represents average glycemic exposure over the preceding two to three months and fails to capture acute stress-induced dysregulation [16–18]. Reliance on either parameter alone may therefore incompletely characterize the true metabolic disturbance during hospitalization.

To overcome these limitations, the Stress Hyperglycemia Ratio (SHR) has been proposed as a composite metric integrating acute and chronic glycemic information. Defined as the ratio of admission glucose to estimated average glucose (eAG) derived from HbA1c, SHR standardizes acute hyperglycemia against baseline glycemic status. By accounting for individual chronic glycemic exposure, SHR more accurately reflects the relative severity of stress-induced hyperglycemia and may serve as a surrogate marker of metabolic reserve and stress tolerance [19,20]. Importantly, SHR provides clinicians with a simple and practical tool to distinguish true stress hyperglycemia from chronically elevated glucose levels, thereby enabling more accurate risk stratification and supporting individualized glycemic management in hospitalized patients. [17,19,20]

Emerging evidence indicates that elevated SHR is associated with increased short-term mortality and adverse events in critically ill patients, as well as those with cardiovascular disease, stroke, and kidney disease [21–23]. Nevertheless, data regarding the association between SHR and mid- to long-term all-cause mortality in hospitalized patients with established T2DM remain limited. Furthermore, the potential nonlinear relationship between SHR and mortality risk has not been fully elucidated. It remains unclear whether a threshold effect exists and whether mortality risk increases proportionally across the entire range of SHR values or predominantly beyond a specific cutoff.

Accordingly, the present study comprehensively investigated the association between SHR and 180-day, 1-year, 3-year, and 5-year all-cause mortality in first-hospitalized patients with T2DM. We further explored potential nonlinear dose–response relationships using restricted cubic spline models, examined the presence of threshold effects, assessed the robustness of the association across clinically relevant subgroups, and evaluated the incremental predictive performance of SHR compared with traditional glycemic parameters. By elucidating the long-term prognostic significance of SHR, this study aims to provide a practical and reliable tool for early risk stratification and individualized management in hospitalized patients with T2DM.

## Methods

### Study population

This retrospective cohort study included adult patients with type 2 diabetes mellitus (T2DM) who were admitted to Beth Israel Deaconess Medical Center (BIDMC), Boston, USA, between 2008 and 2019.The study protocol was approved by the Institutional Review Board (IRB) of BIDMC, and written informed consent was waived due to the retrospective and de-identified data design. This study was also approved with an exemption by the Human Research Ethics Committee of Khon Kaen University, Thailand (Project No. HE691030). The authors obtained legal authorization to use the database (Certificate No. 56073040).

In this study, “first-hospitalized” was defined as the first recorded inpatient admission in the BIDMC database during the study period.

Inclusion criteria were as follows: (1) age ≥ 18 years; (2) diagnosis of T2DM confirmed by clinical and laboratory examinations at first admission. Exclusion criteria were as follows: (1) missing baseline ABG or glycated HbA1c data in the initial laboratory tests; (2) clinically implausible or extreme outlier values in clinical data.

The follow-up endpoint was all-cause mortality, and patients were followed until death or the predefined 5-year follow-up endpoint, whichever occurred first.

Given that calculation of SHR requires both ABG and HbA1c, patients with missing values in either variable were excluded to avoid bias introduced by imputing primary exposure variables.

Based on a data-driven threshold effect analysis, patients were categorized into low and high SHR groups for subsequent analyses.

### Data collection

Baseline clinical characteristics collected at admission included demographic data (age, gender, race), comorbidities (cerebrovascular disease, chronic pulmonary disease, liver disease, cardiovascular disease, renal disease), laboratory biomarkers measured within 24 hours after admission (potential of hydrogen (pH), partial pressure of oxygen (PaO_2_), lactate, white blood cell (WBC), creatinine, blood urea nitrogen (BUN), hematocrit, hemoglobin, platelets, estimated glomerular filtration rate (eGFR), anion gap, bicarbonate, calcium, chloride, sodium, potassium, ABG within 3 hours of admission,HbA1c), and medication use (metformin, basal insulin, insulin).

The primary outcomes were 3-year and 5-year all-cause mortality, and the secondary outcomes were 180-day and 1-year all-cause mortality. The Charlson Comorbidity Index (CCI) score was calculated for all participants for clinical risk stratification. The SHR was calculated as ABG divided by eAG: SHR = ABG/ eAG [18]. The eAG was derived from HbA1c using the A1C-Derived Average Glucose (ADAG) equation: eAG = 28.7 × HbA1c (%) − 46.7 (mg/dL) [24].

### Statistical analysis

All statistical analyses were performed using R software version 4.3.1, with a two-sided significance level of α = 0.05. After normality testing, continuous data were presented as the mean ± standard deviation for normally distributed variables or median (interquartile range) for non-normally distributed variables. Categorical data were expressed as number (percentage), and between-group comparisons were performed using the χ² test or Wilcoxon rank-sum test. Outliers were identified using interquartile range (IQR)-based methods with additional clinical plausibility checks, and biologically implausible laboratory values were excluded. Variables with a missing rate ≥10% were excluded from subsequent analyses to avoid bias from excessive imputation uncertainty. For covariates with a missing proportion <10% (S1 Table), multiple imputation was performed using the multivariate imputation by chained equations (MICE) algorithm under the missing-at-random (MAR) assumption.

A restricted cubic spline (RCS) model was used to analyze the nonlinear association between SHR and 180-day, 1-year, 3-year, and 5-year all-cause mortality, and the likelihood ratio test was used to verify the statistical significance of the nonlinear trend [25]. A piecewise linear regression model was applied for threshold effect analysis to determine the optimal cutoff value for the association between SHR and all-cause mortality at each time point [26], and the significance of the threshold effect was verified by the likelihood ratio test.

The Kaplan-Meier method was used to plot survival curves for patients in different SHR groups stratified by the optimal threshold, and the Log-rank test was used to compare survival differences between groups. Sequentially risk-adjusted Cox proportional hazards regression models were constructed: Model 2 was adjusted for demographic characteristics (sex, race, age); Model 3 was further adjusted for comorbidities (myocardial infarction, congestive heart failure, peripheral vascular disease, cerebrovascular disease, chronic pulmonary disease, renal disease, and liver disease); Model 4 was additionally adjusted for hypoglycemic agents (metformin, insulin, and basal insulin). Hazard ratios (HR) and 95% confidence intervals (CI) were calculated using continuous SHR and categorical SHR as exposure variables to evaluate the independent association between SHR and 3-year and 5-year all-cause mortality.

To assess the robustness of the results, a sensitivity analysis was conducted by excluding patients with severe acute conditions, defined as lactate >4 mmol/L, pH < 7.2, or eGFR < 30 mL/min/1.73 m². The Cox proportional hazards models were then re-estimated in the restricted cohort.

Subgroup analyses were performed to examine the stability of the association between SHR and 3-year and 5-year all-cause mortality in clinical subgroups including myocardial infarction, congestive heart failure, and cerebrovascular disease after adjustment for demographic characteristics. The HR and 95% CI for each subgroup were calculated, and effect heterogeneity across subgroups was assessed using interaction tests.

Time-dependent receiver operating characteristic (ROC) curve analysis was used to evaluate the predictive performance of the models. The multivariate Cox model adjusted for demographics, comorbidities, and hypoglycemic agents was used as the base model. SHR, ABG, and HbA1c were added incrementally to the base model, and the area under the curve (AUC) at 3 and 5 years was calculated. Differences in AUC between different models were compared using the DeLong test. The concordance index (C-index) was also calculated to validate the predictive ability of the models and to assess the incremental predictive value of SHR for mid-to long-term all-cause mortality.

### Ethics statement

This retrospective study was approved by the IRB of BIDMC, Boston, Massachusetts, USA. Written informed consent was waived for the present study due to its retrospective design and the use of de-identified patient data. Additional ethical exemption was granted by the Human Research Ethics Committee of Khon Kaen University, Thailand (Project No.: HE691030). Legal authorization to access and use the study database was obtained by the authors (Certificate No.: 56073040). All procedures were performed in accordance with the Declaration of Helsinki and relevant local ethical regulations.

## Results

### Patient enrollment flowchart, baseline characteristics and between-group comparisons

**Fig 1:**
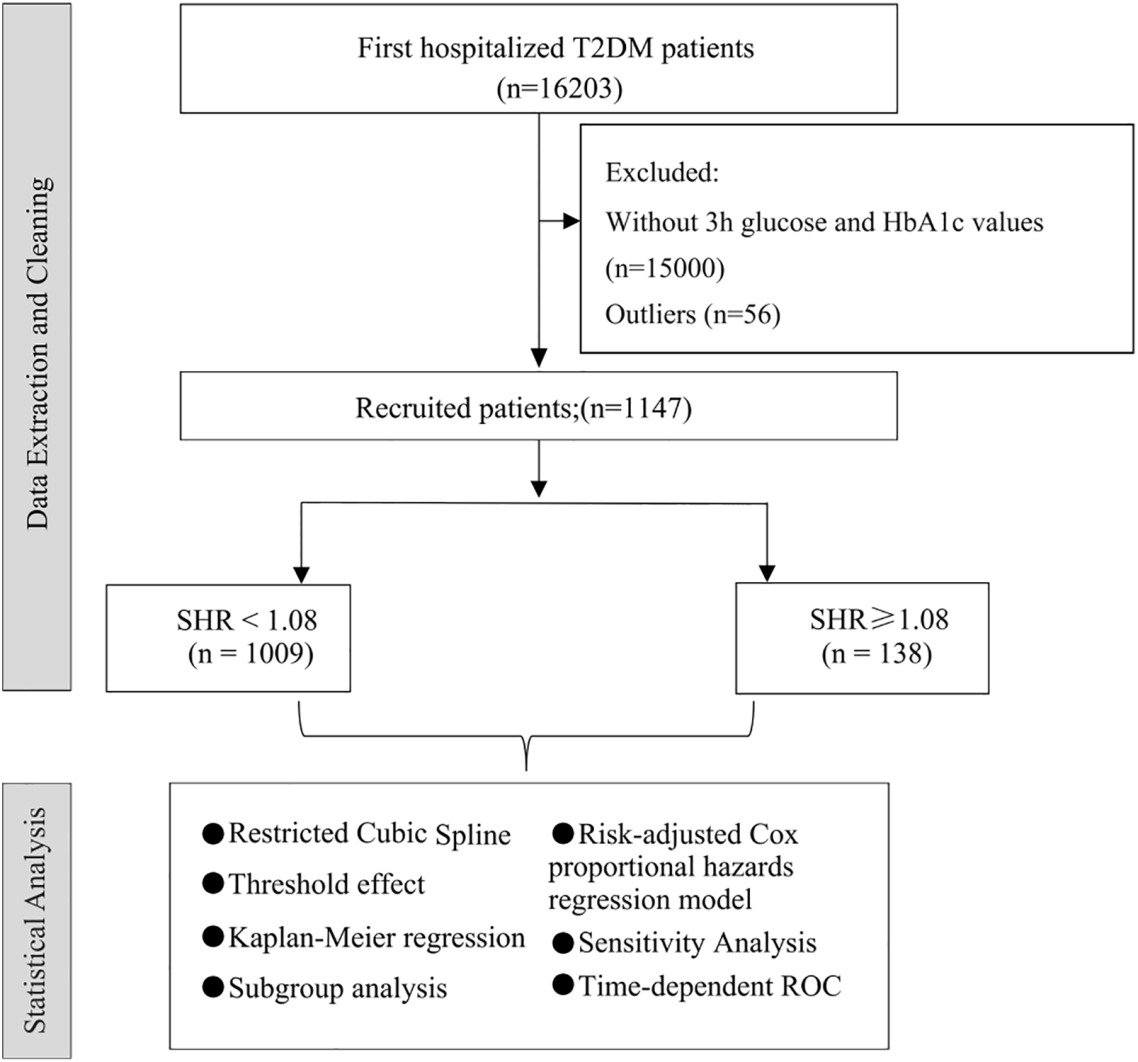
Flow chart of study participant selection. This flow diagram illustrates the inclusion and exclusion process used to identify eligible participants. Among 16,203 initially hospitalized patients with T2DM, individuals with missing baseline glucose or HbA1c data and those with extreme outlier values were excluded. A total of 1,147 patients were ultimately included in the final analysis.

A total of 16,203 initially hospitalized T2DM patients were enrolled in this study. Among them, 15,000 patients were excluded due to missing 3h baseline blood glucose and HbA1c data, and 56 patients were excluded due to outliers. Finally, 1,147 patients were included for subsequent analysis (Fig 1). According to the SHR threshold of 1.08, patients were divided into the SHR < 1.08 group (n = 1009, 87.97%) and the SHR ≥ 1.08 group (n = 138, 12.03%). Comparison of baseline characteristics revealed significant between-group differences in pH, PaO_2_, lactate, WBC, CCI, creatinine, BUN, hematocrit, hemoglobin, platelets, eGFR, anion gap, bicarbonate, calcium, chloride, sodium, potassium, gender, race, cardiovascular disease, renal disease, metformin, basal insulin, 180-day death, 1-year death, 3-year death, and 5-year death (all P < 0.05). However, no significant differences were observed in age, cerebrovascular disease, chronic pulmonary disease, liver disease, or insulin use (all P > 0.05) (Table 1).

**Table 1:** Baseline characteristics of first-admitted patients with type 2 diabetes mellitus.

Variables	Total (n = 1147)	SHR < 1.08 (n = 1009)	SHR ≥ 1.08 (n = 138)	z(χ2)/P
**Demographics**				
Age, median (IQR)	68.00 (61.00, 75.00)	68.00 (61.00, 75.00)	69.00 (59.25, 76.00)	−0.96/0.339
Gender, n (%)				
Female	382 (33.30)	323 (32.01)	59 (42.75)	6.31/0.012
Male	765 (66.70)	686 (67.99)	79 (57.25)	
Race, n (%)				
Asian	45 (3.92)	41 (4.06)	4 (2.90)	15.16/0.004
Black	87 (7.59)	71 (7.04)	16 (11.59)	
Hispanic	43 (3.75)	37 (3.67)	6 (4.35)	
Others	299 (26.07)	249 (24.68)	50 (36.23)	
White	673 (58.67)	611 (60.56)	62 (44.93)	
**Comorbidities, n (%)**				
Myocardial Infarct	290 (25.28)	239 (23.69)	51 (36.96)	11.32/<.001
Congestive Heart Failure	304 (26.50)	249 (24.68)	55 (39.86)	14.36/<.001
Cerebrovascular Disease	118 (10.29)	99 (9.81)	19 (13.77)	2.06/0.151
CPD	220 (19.18)	193 (19.13)	27 (19.57)	0.01/0.903
Renal Disease	294 (25.63)	240 (23.79)	54 (39.13)	14.99/<.001
Liver Disease	103 (8.98)	88 (8.72)	15 (10.87)	0.69/0.408
**Score, median (IQR)**				
Charlson Comorbidity Index	5.00 (4.00, 7.00)	5.00 (4.00, 7.00)	7.00 (5.00, 9.00)	−4.77/<.001
**Laboratory examination, median (IQR)**				
WBC, 10^9^/L	11.80 (8.80, 15.40)	11.80 (8.80, 15.00)	12.30 (9.85, 19.40)	−3.25/0.001
Creatinine, mg/dl	1.00 (0.80, 1.30)	1.00 (0.80, 1.30)	1.40 (1.00, 2.20)	−8.53/<.001
BUN, mg/dl	18.00 (14.00, 26.00)	18.00 (14.00, 25.00)	28.50 (20.00, 40.75)	−8.54/<.001
Hematocrit, %	36.00 (31.80, 40.00)	36.50 (33.00, 40.40)	29.00 (24.92, 34.22)	−10.47/<.001
Hemoglobin, g/dl	10.30 (9.00, 11.70)	10.30 (9.10, 11.90)	9.35 (8.20, 10.85)	−4.88/<.001
Platelets, 10^9^/L	173.0 (133.0, 221.0)	173.0 (133.0, 215.0)	185.0 (134.8, 256.8)	−1.98/0.048
eGFR, mL/min/1.73m²	78.49 (51.98, 96.57)	83.52 (56.48, 97.60)	48.33 (28.60, 66.87)	−8.94/<.001
Anion Gap, mEq/L	13.00 (11.00, 16.00)	13.00 (10.00, 15.00)	16.50 (13.25, 19.00)	−8.59/<.001
Bicarbonate, mEq/L	22.00 (20.00, 24.00)	22.00 (21.00, 24.00)	20.00 (17.00, 22.00)	−7.98/<.001
Calcium, mmol/L	1.14 (1.10,1.19)	1.14 (1.10,1.19)	1.12 (1.03, 1.19)	−2.62/0.009
Chloride, mmol/L	104.0 (101.0, 107.0)	104.0 (102.0, 107.0)	103.0 (99.0, 107.0)	−2.71/0.007
Sodium, mmol/L	137.0 (135.0, 140.0)	137.0 (136.0, 140.0)	136.0 (133.0, 140.0)	−2.56/<.001
Potassium, mmol/L	4.00 (3.70, 4.50)	4.00 (3.70, 4.40)	4.35 (3.90, 5.00)	−5.06/<.001
pH	7.37 (7.32, 7.40)	7.37 (7.33, 7.41)	7.32 (7.24, 7.38)	−6.52/<.001
Pao2, mmHg	242.0 (125.0, 357.5)	256.0 (146.0, 365.0)	126.0 (57.8, 236.3)	−7.82/<.001
Lactate, mmHg	1.70 (1.30, 2.30)	1.60 (1.20, 2.20)	2.70 (1.70, 5.35)	−8.74/<.001
**Drugs, n (%)**				
Metformin	437 (38.10)	417 (41.33)	20 (14.49)	37.07/<.001
Insulin	1095 (95.47)	959 (95.04)	136 (98.55)	3.45/0.063
Basal Insulin	563 (49.08)	537 (53.22)	26 (18.84)	57.42/<.001
**Outcomes, n (%)**				
Death 180d	162 (14.12)	110 (10.90)	52 (37.68)	71.78/<.001
Death 1y	196 (17.09)	138 (13.68)	58 (42.03)	68.88/<.001
Death 3y	218 (19.01)	158 (15.66)	60 (43.48)	61.03/<.001
Death 5y	232 (20.23)	171 (16.95)	61 (44.20)	55.89/<.001

Z: Mann-Whitney test, χ²: Chi-square test, CPD: chronic pulmonary disease, pH: potential of hydrogen, PaO_2_: partial pressure of arterial oxygen, WBC: white blood cell count, BUN: blood urea nitrogen, SHR: stress hyperglycemia ratio, eGFR: estimated glomerular filtration rate.

### Nonlinear association and threshold effect of SHR with all-cause mortality

**Fig 2:**
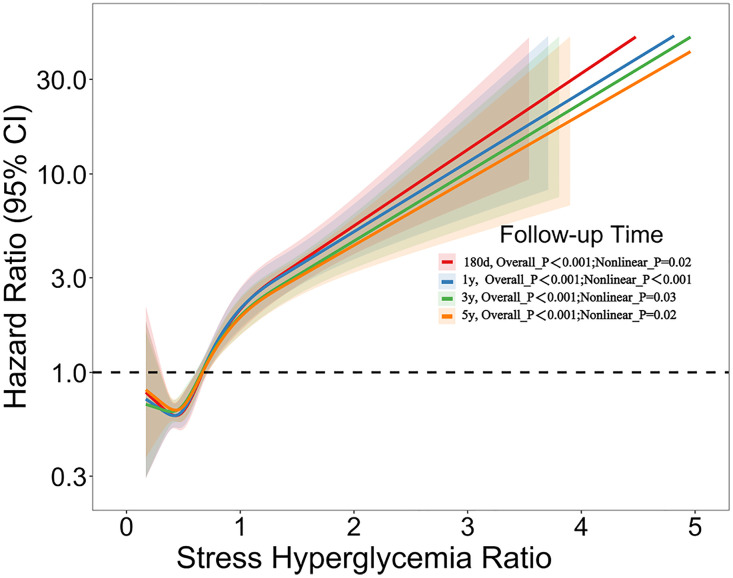
Nonlinear association between stress hyperglycemia ratio and mortality. Two-piecewise threshold effect models (Fig 3) identified inflection values of 1.29 for 180-day, 1.07 for 1-year, and 1.08 for 3-year and 5-year mortality, all of which were statistically validated by likelihood ratio tests (180-day P = 0.028; 1-year P = 0.0093; 3-year P = 0.029; 5-year P = 0.042). For 3-year mortality, the slope of the exposure–response curve was steeper below the inflection point of 1.08 (HR = 6.83, 95% CI: 3.29–14.16), while risk remained significantly elevated above this threshold (HR = 2.17, 95% CI: 1.39–3.38). Consistent results were observed for 5-year mortality (SHR < 1.08: HR = 5.85, 95% CI: 2.86–11.95, P < 0.001; SHR ≥ 1.08: HR = 2.05, 95% CI: 1.31–3.21, P = 0.002). Importantly, the two segments reflect differences in the slope of risk increase rather than differences in absolute risk levels. The steeper slope below 1.08 indicates a greater rate of risk increase per unit change in SHR within the lower range.

RCS demonstrated significant nonlinear associations between SHR and all-cause mortality across all follow-up periods (Fig 2), indicating a non-uniform dose–response relationship rather than a constant linear effect.

### Survival analysis across SHR groups

**Fig 3:**
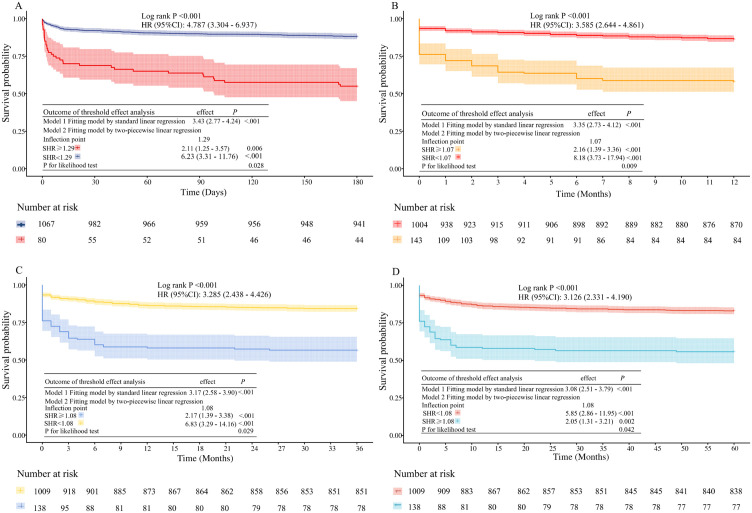
Kaplan–Meier survival curves stratified by SHR threshold. Kaplan–Meier curves showing cumulative incidence of all-cause mortality at 180 days, 1 year, 3 years, and 5 years among first-hospitalized patients with type 2 diabetes mellitus, stratified by SHR threshold (1.08). Log-rank tests were used to compare survival differences between groups. Numbers at risk at sequential follow-up time points are shown.

Patients were categorized into low SHR (<1.08, n = 1009) and high SHR (≥1.08, n = 138) groups. Kaplan–Meier survival analysis (Fig 3) showed significantly higher 180-day, 1-year, 3-year, and 5-year all-cause mortality in the high SHR group compared with the low SHR group (log-rank P < 0.001 for all). The unadjusted HRs were 3.17 (95% CI: 2.58–3.90) for 3-year mortality and 3.08 (95% CI: 2.51–3.79) for 5-year mortality. The number at risk decreased progressively over time in both groups, with a faster reduction observed in the high SHR group.

### Subgroup analysis

**Fig 4:**
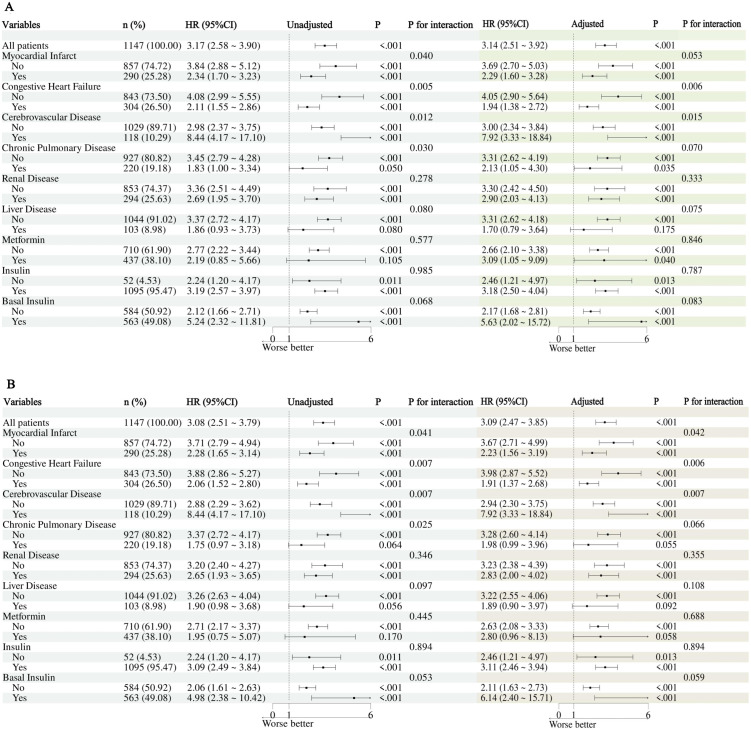
Subgroup analysis of the association between SHR and mortality risk. Subgroup analyses evaluating the association between stress hyperglycemia ratio and all-cause mortality at 3 years (upper panel) and 5 years (lower panel) in first-hospitalized patients with type 2 diabetes mellitus. Hazard ratios (95% confidence intervals) were estimated after adjustment for age, sex, and race. Interaction tests were performed to assess heterogeneity across clinical subgroups.

Subgroup analyses (Fig 4) using SHR as a continuous variable showed that after adjustment for age, sex, and race, the association between elevated SHR and increased 3-year and 5-year mortality risk remained consistent across most clinical subgroups. For 3-year mortality, the adjusted HR was 3.14 (95% CI: 2.51–3.92, P < 0.001). Significant interactions were observed for congestive heart failure (P = 0.006) and cerebrovascular disease (P = 0.015), while myocardial infarction (P for interaction = 0.053), chronic pulmonary disease (P = 0.070), and baseline insulin use (P = 0.083) showed borderline interactions. No significant interactions were observed for renal disease, liver disease, metformin use, or insulin use. For 5-year mortality, the adjusted HR was 3.09 (95% CI: 2.47–3.85, P < 0.001), with significant interactions for myocardial infarction (P = 0.042), congestive heart failure (P = 0.006), and cerebrovascular disease (P = 0.007), while baseline insulin use remained borderline (P = 0.059). These results indicate that the association between SHR and mid-to-long-term mortality is robust overall and is significantly modified by underlying cardiovascular and cerebrovascular comorbidities.

### Sequential risk-adjusted Cox proportional hazards analysis and sensitivity analysis

Sequentially adjusted Cox models (Tables 2 and 3) demonstrated that SHR remained significantly associated with 3-year and 5-year mortality across all models, including unadjusted, demographic-adjusted, comorbidity-adjusted, and fully adjusted models (all P < 0.001). In the fully adjusted model, each 0.1-unit increase in SHR was associated with HR = 1.10 (95% CI: 1.08–1.13) for 3-year mortality and HR = 1.10 (95% CI: 1.07–1.13) for 5-year mortality. Stratified analysis using 1.08 as the cutoff showed that SHR ≥ 1.08 consistently conferred higher mortality risk across all models (P < 0.001), with HR = 2.38 (95% CI: 1.73–3.27) for 3-year mortality and HR = 2.28 (95% CI: 1.67–3.12) for 5-year mortality in the fully adjusted model, indicating that SHR is an independent predictor of long-term mortality in first-hospitalized T2DM patients.

**Table 2:** Association between per 0.1 unit increment in SHR and 3-year and 5-year all-cause mortality (sequentially adjusted Cox models).

Variables	Model 1		Model 2		Model 3		Model 4	
**HR (95%CI)**	**P**	**HR (95%CI)**	**P**	**HR (95%CI)**	**P**	**HR (95%CI)**	**P**
SHR-3 years	1.12 (1.10–1.14)	<.001	1.12 (1.10–1.14)	<.001	1.11 (1.09–1.13)	<.001	1.10 (1.08–1.13)	<.001
SHR-5 years	1.12 (1.10–1.14)	<.001	1.12 (1.10–1.14)	<.001	1.11 (1.09–1.13)	<.001	1.10 (1.07–1.13)	<.001

HR: Hazard Ratio, CI: Confidence Interval.

**Table 3:** SHR stratification and clinical outcomes in T2DM (sequentially adjusted Cox models).

Variables	Model 1^a^		Model 2^b^		Model 3^c^		Model 4^d^	
**HR (95%CI)**	**P**	**HR (95%CI)**	**P**	**HR (95%CI)**	**P**	**HR (95%CI)**	**P**
3 years								
SHR < 1.08	1.00 (Reference)		1.00 (Reference)		1.00 (Reference)		1.00 (Reference)	
SHR ≥ 1.08	3.49 (2.59 – 4.69)	<.001	3.14 (2.32 – 4.25)	<.001	2.66 (1.94 – 3.64)	<.001	2.38 (1.73 – 3.27)	<.001
5 years								
SHR < 1.08	1.00 (Reference)		1.00 (Reference)		1.00 (Reference)		1.00 (Reference)	
SHR ≥ 1.08	3.31 (2.47 – 4.43)	<.001	3.04 (2.26 – 4.10)	<.001	2.56 (1.88 – 3.49)	<.001	2.28 (1.67– 3.12)	<.001

HR: Hazard Ratio, CI: Confidence Interval.

^a^Model 1: Crude.

^b^Model 2: Adjust: gender, race, age.

^c^Model 3: Adjust: Model 2 + myocardial infarct, congestive heart failure, peripheral vascular disease, cerebrovascular disease, chronic pulmonary disease, renal disease, liver disease.

^d^Model 4: Adjust: Model 2 + Model 3 + metformin, insulin.

To examine whether the observed association was influenced by severe acute illness, a sensitivity analysis was conducted after excluding patients with lactate >4 mmol/L, pH < 7.2, or eGFR < 30 mL/min/1.73m² (S2 Table). SHR remained significantly associated with increased long-term mortality when analyzed as a continuous variable. In the fully adjusted model, each 0.1-unit increase in SHR was associated with a higher risk of both 3-year mortality (HR = 1.15, 95% CI: 1.09–1.20, P < 0.001) and 5-year mortality (HR = 1.14, 95% CI: 1.09–1.19, P < 0.001).

Consistent results were observed when SHR was analyzed as a categorical variable based on the identified threshold value of 1.08 (S3 Table). Compared with patients with SHR < 1.08, those with SHR ≥ 1.08 had significantly higher risks of 3-year mortality (adjusted HR = 2.92, 95% CI: 1.76–4.86, P < 0.001) and 5-year mortality (adjusted HR = 2.75, 95% CI: 1.68–4.50, P < 0.001). These findings indicate that the association between SHR and long-term mortality remained robust after excluding critically ill patients.

### Time-dependent predictive performance and incremental value of SHR

**Fig 5:**
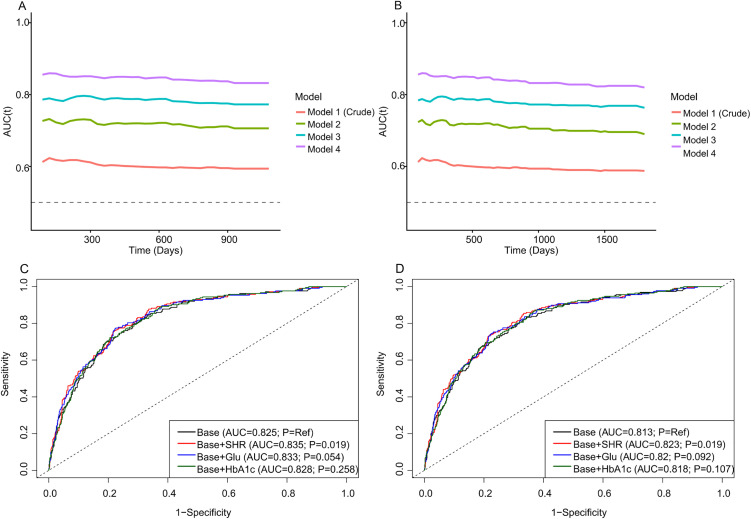
Predictive performance of SHR for mortality risk. **(A, B)** Time-dependent ROC curves showing predictive performance of sequentially adjusted models for 3-year (A) and 5-year (B) all-cause mortality. **(C, D)** Incremental predictive value of stress hyperglycemia ratio compared with admission glucose and HbA1c when added to the base model including demographics, comorbidities, and hypoglycemic agents. Area under the curve (AUC) values and DeLong test P values are presented.

Time-dependent receiver operating characteristic (ROC) analysis (Figs 5A and 5B) showed that the fully adjusted model (Model 4) maintained strong predictive performance for both 3-year and 5-year all-cause mortality, with AUC values consistently above 0.80 during follow-up. For 3-year mortality, Model 4 achieved AUC values of approximately 0.85–0.86, outperforming Model 1 (~0.62), Model 2 (~0.72), and Model 3 (~0.80). A similar pattern was observed for 5-year mortality, where Model 4 maintained AUC values of approximately 0.84–0.85.

Incremental predictive analyses (Figs 5C and 5D) further assessed the incremental prognostic contribution of SHR beyond conventional glycemic indicators. For 3-year mortality, the base model yielded an AUC of 0.825, which increased to 0.835 after adding SHR (P = 0.019). In comparison, addition of ABG or HbA1c resulted in minimal and non-significant changes in discrimination (AUC = 0.833, P = 0.054; AUC = 0.828, P = 0.258, respectively).

Similarly, for 5-year mortality, the AUC increased from 0.813 to 0.823 after incorporating SHR (P = 0.019), whereas no significant improvement was observed after adding ABG or HbA1c individually.

## Discussion

This study demonstrates that the SHR is independently associated with mid- to long-term all-cause mortality in first-hospitalized patients with T2DM, with a non-linear relationship and a threshold effect observed around 1.08. These findings suggest that SHR may capture not only stress-related hyperglycemia but also heterogeneity in metabolic vulnerability under acute physiological stress. Importantly, its prognostic relevance appears to extend beyond short-term critical illness, indicating potential long-term risk stratification value.

Building on its clinical relevance, stress‐induced glycemic dysregulation has increasingly been recognized as an important determinant of outcomes in hospitalized patients with acute and chronic conditions. Unlike ABG or HbA1c alone, SHR integrates acute and chronic glycemic status by standardizing ABG against eAG, thereby potentially providing a more balanced reflection of stress-related metabolic perturbation while reducing dependence on baseline glycemia. [17–19,27–33].

Previous studies have primarily focused on short-term outcomes in critically ill patients, acute coronary syndrome, and stroke, where SHR has been associated with in-hospital or 30-day mortality [17,21–23,34–38]. However, most of these studies assumed a linear relationship and were limited by short follow-up periods. In contrast, our findings extend these observations to first-hospitalized patients with T2DM and suggest that the association between SHR and mortality may persist over a longer time horizon (up to 5 years). We further observed evidence of a non-linear pattern with a threshold effect, indicating that the relationship between stress-related dysglycemia and outcomes may be more complex than previously appreciated.

Restricted cubic spline and threshold analyses suggested a non-linear association between SHR and mortality, with heterogeneous risk gradients across exposure levels. The steeper slope observed below 1.08 may reflect greater sensitivity to metabolic stress within this range, whereas the association above this threshold remained significant but less pronounced. These findings should be interpreted as reflecting differences in risk gradients rather than absolute risk separation.

Although ABG reflects acute status and HbA1c reflects long-term glycemic exposure, both have inherent limitations when considered independently. SHR, by combining these measures, may better reflect relative stress-induced glycemic deviation while partially accounting for baseline glycemic status [18,19]. In this study, models incorporating SHR showed modest but statistically significant improvements in discrimination compared with models using ABG or HbA1c alone, suggesting that SHR may provide incremental prognostic information beyond traditional glycemic indicators.

Survival analysis and sequentially risk‐adjusted Cox regression further confirmed the independent prognostic value of SHR. In this study, the SHR ≥ 1.08 group had significantly higher mortality at all follow‐up time points, and the association remained highly significant after sequential adjustment for demographics, cardiocerebrovascular, hepatic and renal comorbidities, and hypoglycemic agents. Each 0.1-unit increase in continuous SHR was associated with approximately 10% higher risks of both 3-year and 5-year mortality. These results are consistent with studies in critically ill and cardiovascular disease populations, but this study extends the evidence to first-hospitalized T2DM patients and highlights the unique value of SHR in the long-term management of metabolic diseases.

Sensitivity analysis was performed by excluding critically ill patients, defined as those with lactate >4 mmol/L, pH < 7.2, or eGFR < 30 mL/min/1.73 m². In this restricted cohort, SHR remained significantly associated with both 3-year and 5-year all-cause mortality, whether analyzed as a continuous variable or categorized using the threshold of 1.08. Notably, the effect estimates were slightly increased after exclusion of critically ill patients. This phenomenon may be explained by reduced confounding from acute physiological instability, competing risks, and treatment-related heterogeneity in the full cohort. In critically ill patients, multiple non-glycemic drivers of mortality may attenuate the relative contribution of stress-related dysglycemia, leading to underestimation of the SHR–mortality association.

This finding suggests that the observed association is unlikely to be fully explained by acute critical illness, and instead reflects a consistent relationship between stress-related dysglycemia and long-term mortality risk. These results further support the robustness and generalizability of SHR as an independent prognostic marker in first-hospitalized T2DM patients.

Subgroup analysis revealed that pre‐existing myocardial infarction, congestive heart failure, and cerebrovascular disease amplified the association between SHR and mortality. Possible mechanisms include chronic inflammation, impaired endothelial function, and oxidative stress inherent to these conditions, which render patients more susceptible to inflammatory cascades and organ hypoperfusion under hospitalization stress. Cardiac or neurological dysregulation also weakens compensatory capacity against metabolic disturbance, which may be linked to more severe clinical outcomes with the same magnitude of relative hyperglycemia [39–42]. Furthermore, patients with cardiocerebrovascular comorbidities often receive multiple medications and have complex comorbidities, which may alter metabolic and inflammatory responses during stress. These findings suggest heightened clinical vigilance for first‐hospitalized T2DM patients with cardiocerebrovascular diseases: even mild relative hyperglycemia may predict poorer long‐term prognosis, warranting more proactive monitoring and intervention strategies.

This study has notable strengths. First, focusing on first‐hospitalized T2DM patients allows SHR to more reliably reflect baseline metabolic reserve and acute stress response. Second, this study did not only confirm the independent association between SHR and long-term mortality but also systematically revealed its nonlinear characteristics using RCS and threshold analyses, addressing the limitation of previous studies based on linear assumptions. Finally, sequential risk adjustment, subgroup analyses, time‐dependent ROC, and incremental predictive analysis were applied to comprehensively evaluate the stability and incremental predictive value of SHR, enhancing the clinical interpretability of the results.

This study also has several limitations. First, this was a single-center retrospective cohort study based on electronic health records. A substantial proportion of patients were excluded due to missing key laboratory data required for the calculation of SHR, which may introduce selection bias and limit the generalizability of the findings. However, this exclusion was necessary to ensure accurate exposure definition and to avoid bias introduced by imputing primary exposure variables. Therefore, external validation in multicenter prospective studies is warranted. Second, only baseline SHR was analyzed, and the prognostic impact of dynamic changes in SHR was not explored; future studies may investigate the relationship between SHR trends and long-term outcomes. Third, although multiple confounders were adjusted for, residual confounding from unmeasured factors such as nutritional status, inflammatory markers, and intensity of in‐hospital treatment cannot be completely excluded. Accordingly, causal inferences cannot be made based on this observational study design. Finally, the assessment of incremental predictive performance was primarily based on changes in AUC. Although SHR showed statistically significant but only limited improvements in discrimination, the magnitude of AUC increase was small. Therefore, these findings should be interpreted as suggesting a complementary rather than substantial improvement in predictive performance. More comprehensive reclassification metrics such as Net Reclassification Improvement and Integrated Discrimination Improvement were not performed due to the retrospective design and the study’s primary focus on model discrimination rather than clinical decision reclassification. Future studies incorporating these metrics are warranted to further evaluate the potential clinical value of SHR.

### Implications for clinical and primary care practice

The findings of this study have important implications across both hospital-based and community-based care settings. Within the inpatient context, the SHR provides a simple and cost-neutral metric derived from routinely available admission glucose and HbA1c values. Identification of a nonlinear association and a threshold around 1.08 suggests that even moderate degrees of relative stress hyperglycemia may reflect substantial metabolic vulnerability. Clinicians may therefore consider elevated SHR as a marker warranting closer inpatient monitoring, comprehensive evaluation of cardiometabolic comorbidities, and careful discharge planning.

Beyond the hospital setting, first hospitalization in patients with T2DM often represents a critical transition point in the trajectory of chronic disease. Following discharge, long-term management is typically coordinated within primary care. Incorporating SHR into discharge summaries may assist general practitioners in identifying individuals at higher risk of adverse outcomes who may benefit from intensified follow-up, earlier medication optimization, stricter cardiovascular risk management, and multidisciplinary support. In particular, patients with concomitant cardiocerebrovascular disease and elevated SHR may require closer longitudinal surveillance given their amplified risk profile.

Importantly, SHR reflects relative metabolic stress rather than absolute hyperglycemia alone, aligning with a more individualized approach to chronic disease management. While the incremental improvement in predictive discrimination was modest, the feasibility and simplicity of SHR calculation support its potential role as a complementary risk stratification tool during care transitions. Prospective studies are needed to determine whether SHR-informed management strategies across hospital and primary care settings can improve long-term clinical outcomes.

## Conclusions

This study demonstrates that SHR is significantly and nonlinearly associated with mid- to long-term all-cause mortality in first-hospitalized T2DM patients, with a threshold of approximately 1.08 for 3-year and 5-year mortality. SHR is an independent prognostic factor for long-term mortality and significantly improves mortality risk prediction beyond a baseline model with conventional covariates, outperforming admission glucose or HbA1c alone. Myocardial infarction, congestive heart failure, and cerebrovascular disease significantly modify the SHR–prognosis relationship, indicating that relative stress hyperglycemia deserves greater clinical attention in patients with cardiocerebrovascular comorbidities. Risk stratification and individualized management strategies based on SHR hold important clinical potential in first-hospitalized T2DM patients. Future prospective, multicenter validation and targeted intervention studies are warranted to further confirm its clinical value.

## Supporting information

S1 Table Missing data pattern of study variables. (DOCX)

S2 Table Sensitivity analysis of the association between per 0.1 unit increment in SHR and mortality after excluding critically ill patients. (DOCX)

S3 Table Sensitivity analysis of the association between categorized SHR and mortality after excluding critically ill patients. (DOCX)

## References

[1] Hossain MJ, Al-Mamun M, Islam MR. Diabetes mellitus, the fastest growing global public health concern: early detection should be focused. Health Sci Rep. 2024;7(3):e2004. doi: 10.1002/hsr2.2004 38524769 PMC10958528

[2] GBD 2021 Diabetes Collaborators. Global, regional, and national burden of diabetes from 1990 to 2021, with projections of prevalence to 2050: a systematic analysis for the Global Burden of Disease Study 2021. Lancet. 2023;402(10397):203–34. doi: 10.1016/S0140-6736(23)01301-6 37356446 PMC10364581

[3] Khan MAB, Hashim MJ, King JK, Govender RD, Mustafa H, Al Kaabi J. Epidemiology of type 2 diabetes - global burden of disease and forecasted trends. J Epidemiol Glob Health. 2020;10(1):107–11. doi: 10.2991/jegh.k.191028.001 32175717 PMC7310804

[4] Cho NH, Shaw JE, Karuranga S, Huang Y, da Rocha Fernandes JD, Ohlrogge AW, et al. IDF diabetes atlas: global estimates of diabetes prevalence for 2017 and projections for 2045. Diabetes Res Clin Pract. 2018;138:271–81. doi: 10.1016/j.diabres.2018.02.023 29496507

[5] Sun H, Saeedi P, Karuranga S, Pinkepank M, Ogurtsova K, Duncan BB, et al. IDF diabetes atlas: global, regional and country-level diabetes prevalence estimates for 2021 and projections for 2045. Diabetes Res Clin Pract. 2022;183:109119. doi: 10.1016/j.diabres.2021.109119 34879977 PMC11057359

[6] American Diabetes Association Professional Practice Committee. 4. Comprehensive Medical Evaluation and Assessment of Comorbidities: Standards of Care in Diabetes-2024. Diabetes Care. 2024;47(Suppl 1):S52–76. doi: 10.2337/dc24-S004 38078591 PMC10725809

[7] American Diabetes Association Professional Practice Committee. Classification and diagnosis of diabetes: Standards of medical care in diabetes-2022. Diabetes Care. 2022;45(Suppl 1):S17–38. doi: 10.2337/dc22-S002 34964875

[8] Saeedi P, Petersohn I, Salpea P, Malanda B, Karuranga S, Unwin N, et al. Global and regional diabetes prevalence estimates for 2019 and projections for 2030 and 2045: results from the International Diabetes Federation Diabetes Atlas, 9th edition. Diabetes Res Clin Pract. 2019;157:107843. doi: 10.1016/j.diabres.2019.107843 31518657

[9] Davies MJ, Aroda VR, Collins BS, et al. Management of hyperglycemia in type 2 diabetes, 2022. A consensus report by the American Diabetes Association (ADA) and the European Association for the Study of Diabetes (EASD). Diabetes Care. 2022;45(11):2753–86. doi: 10.2337/dci22-0034 36148880 PMC10008140

[10] ElSayed NA, Aleppo G, Aroda VR. Glycemic targets: standards of care in diabetes-2023. Diabetes Care. 2023;46(Suppl 1):S97–110. doi: 10.2337/dc23-S006 PMC9810469 36507646

[11] American Diabetes Association Professional Practice Committee for Diabetes. Diabetes care in the hospital: standards of care in diabetes-2026. Diabetes Care. 2026;49(Supplement_1):S339–55. doi: 10.2337/dc26-S016 PMC12690180 41358892

[12] Caturano A, Rocco M, Tagliaferri G, Piacevole A, Nilo D, Di Lorenzo G, et al. Oxidative stress and cardiovascular complications in type 2 diabetes: from pathophysiology to lifestyle modifications. Antioxidants (Basel). 2025;14(1):72. doi: 10.3390/antiox14010072 39857406 PMC11759781

[13] An Y, Xu B-T, Wan S-R, Ma X-M, Long Y, Xu Y, et al. The role of oxidative stress in diabetes mellitus-induced vascular endothelial dysfunction. Cardiovasc Diabetol. 2023;22(1):237. doi: 10.1186/s12933-023-01965-7 37660030 PMC10475205

[14] Cao B, Guo Z, Li D-T, Zhao L-Y, Wang Z, Gao Y-B, et al. The association between stress-induced hyperglycemia ratio and cardiovascular events as well as all-cause mortality in patients with chronic kidney disease and diabetic nephropathy. Cardiovasc Diabetol. 2025;24(1):55. doi: 10.1186/s12933-025-02610-1 39915833 PMC11803992

[15] Alabdali MM, Alrasheed AS, Alghirash FA, Almaqboul TM, Alhashim A, Aljaafari DT, et al. Stress hyperglycemia as a prognostic indicator of the clinical outcomes in patients with stroke: a comprehensive literature review. Biomedicines. 2025;13(8):1834. doi: 10.3390/biomedicines13081834 40868089 PMC12383850

[16] Chen T, Zhu Y, Liu Y, Li H, Han Z, Liu M, et al. Stress hyperglycemia ratio: a novel prognostic marker in chronic kidney disease. Diabetol Metab Syndr. 2025;17(1):69. doi: 10.1186/s13098-025-01639-2 40001175 PMC11854335

[17] Li X-H, Yang X-L, Dong B-B, Liu Q. Predicting 28-day all-cause mortality in patients admitted to intensive care units with pre-existing chronic heart failure using the stress hyperglycemia ratio: a machine learning-driven retrospective cohort analysis. Cardiovasc Diabetol. 2025;24(1):10. doi: 10.1186/s12933-025-02577-z 39780223 PMC11714879

[18] Roberts GW, Quinn SJ, Valentine N, Alhawassi T, O’Dea H, Stranks SN, et al. Relative hyperglycemia, a marker of critical illness: introducing the stress hyperglycemia ratio. J Clin Endocrinol Metab. 2015;100(12):4490–7. doi: 10.1210/jc.2015-2660 26485219

[19] Dungan KM, Braithwaite SS, Preiser J-C. Stress hyperglycaemia. Lancet. 2009;373(9677):1798–807. doi: 10.1016/S0140-6736(09)60553-5 19465235 PMC3144755

[20] Huang Y-W, An Y-H, Yin X-S, Li Z-P. Association of the stress hyperglycemia ratio and clinical outcomes in patients with cardiovascular diseases: a systematic review and meta-analysis. Eur Rev Med Pharmacol Sci. 2022;26(24):9258–69. doi: 10.26355/eurrev_202212_30679 36591838

[21] Tan M-Y, Zhang Y-J, Zhu S-X, Wu S, Zhang P, Gao M. The prognostic significance of stress hyperglycemia ratio in evaluating all-cause and cardiovascular mortality risk among individuals across stages 0-3 of cardiovascular-kidney-metabolic syndrome: evidence from two cohort studies. Cardiovasc Diabetol. 2025;24(1):137. doi: 10.1186/s12933-025-02689-6 40128747 PMC11934678

[22] Esdaile H, Khan S, Mayet J, Oliver N, Reddy M, Shah ASV. The association between the stress hyperglycaemia ratio and mortality in cardiovascular disease: a meta-analysis and systematic review. Cardiovasc Diabetol. 2024;23(1):412. doi: 10.1186/s12933-024-02454-1 39550575 PMC11568630

[23] Ali Abdelhamid Y, Kar P, Finnis ME, Phillips LK, Plummer MP, Shaw JE, et al. Stress hyperglycaemia in critically ill patients and the subsequent risk of diabetes: a systematic review and meta-analysis. Crit Care. 2016;20(1):301. doi: 10.1186/s13054-016-1471-6 27677709 PMC5039881

[24] Nathan DM, Kuenen J, Borg R, Zheng H, Schoenfeld D, Heine RJ, et al. Translating the A1C assay into estimated average glucose values. Diabetes Care. 2008;31(8):1473–8. doi: 10.2337/dc08-0545 18540046 PMC2742903

[25] Desquilbet L, Mariotti F. Dose-response analyses using restricted cubic spline functions in public health research. Stat Med. 2010;29(9):1037–57. doi: 10.1002/sim.3841 20087875

[26] Muggeo VMR. Estimating regression models with unknown break-points. Stat Med. 2003;22(19):3055–71. doi: 10.1002/sim.1545 12973787

[27] Yan F, Chen X, Quan X, Wang L, Wei X, Zhu J. Association between the stress hyperglycemia ratio and 28-day all-cause mortality in critically ill patients with sepsis: a retrospective cohort study and predictive model establishment based on machine learning. Cardiovasc Diabetol. 2024;23(1):163. doi: 10.1186/s12933-024-02265-4 38725059 PMC11084034

[28] Cheng S, Shen H, Han Y, Han S, Lu Y. Association between stress hyperglycemia ratio index and all-cause mortality in critically ill patients with atrial fibrillation: a retrospective study using the MIMIC-IV database. Cardiovasc Diabetol. 2024;23(1):363. doi: 10.1186/s12933-024-02462-1 39402588 PMC11476318

[29] Li L, Zhao M, Zhang Z, Zhou L, Zhang Z, Xiong Y, et al. Prognostic significance of the stress hyperglycemia ratio in critically ill patients. Cardiovasc Diabetol. 2023;22(1):275. doi: 10.1186/s12933-023-02005-0 37833697 PMC10576399

[30] Wang F, Guo Y, Tang Y, Zhao S, Xuan K, Mao Z, et al. Combined assessment of stress hyperglycemia ratio and glycemic variability to predict all-cause mortality in critically ill patients with atherosclerotic cardiovascular diseases across different glucose metabolic states: an observational cohort study with machine learning. Cardiovasc Diabetol. 2025;24(1):199. doi: 10.1186/s12933-025-02762-0 40346649 PMC12065353

[31] Chen Y, Xu J, He F, Huang A, Wang J, Liu B, et al. Assessment of stress hyperglycemia ratio to predict all-cause mortality in patients with critical cerebrovascular disease: a retrospective cohort study from the MIMIC-IV database. Cardiovasc Diabetol. 2025;24(1):58. doi: 10.1186/s12933-025-02613-y 39920777 PMC11806754

[32] Li X, Qiao Y, Ruan L, Xu S, Fan Z, Liu S, et al. Stress hyperglycemia ratio as an independent predictor of acute kidney injury in critically ill patients with acute myocardial infarction: a retrospective U.S. cohort study. Ren Fail. 2025;47(1):2471018. doi: 10.1080/0886022X.2025.2471018 40012169 PMC11869341

[33] Tian J, Zhou T, Liu Z, Dong Y, Xu H. Stress hyperglycemia is associated with poor prognosis in critically ill patients with cardiogenic shock. Front Endocrinol (Lausanne). 2024;15:1446714. doi: 10.3389/fendo.2024.1446714 39301321 PMC11410614

[34] Chen X, Yang Z, Shi R, Wang X, Li X. Stress hyperglycemia ratio association with all-cause mortality in critically ill patients with coronary heart disease: an analysis of the MIMIC-IV database. Sci Rep. 2024;14(1):29110. doi: 10.1038/s41598-024-80763-x 39582018 PMC11586423

[35] Song G, Liu X, Lu Z, Guan J, Chen X, Li Y, et al. Relationship between stress hyperglycaemic ratio (SHR) and critical illness: a systematic review. Cardiovasc Diabetol. 2025;24(1):188. doi: 10.1186/s12933-025-02751-3 40317019 PMC12049067

[36] Chen L, Zeng X, Zou W, Chen M, Fan Y, Huang P. Predictive performance of stress hyperglycemia ratio for poor prognosis in critically ill patients: a systematic review and dose-response meta-analysis. Eur J Med Res. 2025;30(1):613. doi: 10.1186/s40001-025-02868-x 40646634 PMC12247285

[37] Zhou Y, Liu L, Huang H, Li N, He J, Yao H, et al. Stress hyperglycemia ratio and in-hospital prognosis in non-surgical patients with heart failure and type 2 diabetes. Cardiovasc Diabetol. 2022;21(1):290. doi: 10.1186/s12933-022-01728-w 36572923 PMC9791974

[38] Xia Z, Gu T, Zhao Z, Xing Q, Zhang Y, Zhang Z, et al. The stress hyperglycemia ratio, a novel index of relative hyperglycemia, predicts short-term mortality in critically ill patients after esophagectomy. J Gastrointest Oncol. 2022;13(1):56–66. doi: 10.21037/jgo-22-11 35284100 PMC8899743

[39] Zhang C, Shen H-C, Liang W-R, Ning M, Wang Z-X, Chen Y, et al. Relationship between stress hyperglycemia ratio and allcause mortality in critically ill patients: results from the MIMIC-IV database. Front Endocrinol (Lausanne). 2023;14:1111026. doi: 10.3389/fendo.2023.1111026 37077351 PMC10106677

[40] Zhou Q, Yang J, Wang W, Shao C, Hua X, Tang Y-D. The impact of the stress hyperglycemia ratio on mortality and rehospitalization rate in patients with acute decompensated heart failure and diabetes. Cardiovasc Diabetol. 2023;22(1):189. doi: 10.1186/s12933-023-01908-2 37495967 PMC10373236

[41] Zhang J, Zhang Q, Gu H, Zhou Q, Li Z, Zhao X. Comparison of stress hyperglycemia ratio and glycemic gap on acute ICH in-hospital outcomes. Ann Clin Transl Neurol. 2024;11(6):1492–501. doi: 10.1002/acn3.52063 38590111 PMC11187964

[42] Yang B, Chen X, Li F, Zhang J, Dong D, Ou H, et al. Stress hyperglycemia increases short-term mortality in acute ischemic stroke patients after mechanical thrombectomy. Diabetol Metab Syndr. 2024;16(1):32. doi: 10.1186/s13098-024-01272-5 38297321 PMC10829332

