# Supplementary material for: Stress hyperglycemia ratio predicts mid- to long-term mortality in first-hospitalized type 2 diabetes: Nonlinear threshold and prognostic value
Source: PLoS One. 2026 Jun 30;21(6):e0351307. doi: 10.1371/journal.pone.0351307 (PMC13318022; doi:10.1371/journal.pone.0351307)
Supplement: S1 Table — (DOCX) [file pone.0351307.s001.docx]

**S1 Table. Missing data pattern of study variables.**

| **Category** | **Variable** | **Missing n^a^** | **Missing %^b^** |
| --- | --- | --- | --- |
| Laboratory | Anion gap | 78 | **6.80** |
| Laboratory | WBC | 69 | **6.02** |
| Laboratory | BUN | 69 | **6.02** |
| Laboratory | Hemoglobin | 69 | **6.02** |
| Laboratory | Platelets | 69 | **6.02** |
| Laboratory | Creatinine | 65 | **5.67** |
| Laboratory | Bicarbonate | 57 | **4.97** |
| Laboratory | Lactate | 24 | **2.09** |
| Laboratory | PaO₂ | 23 | **2.01** |
| Laboratory | pH | 18 | **1.57** |
| Laboratory | Calcium | 7 | **0.61** |
| Laboratory | Chloride | 2 | **0.17** |
| Laboratory | Sodium | 2 | **0.17** |

WBC, white blood cell; BUN, blood urea nitrogen; PaO₂, partial pressure of arterial oxygen. ^a^Missing n, number of missing cases; ^b^Missing %, percentage of missing values.
