# Supplementary material for: Stress hyperglycemia ratio predicts mid- to long-term mortality in first-hospitalized type 2 diabetes: Nonlinear threshold and prognostic value
Source: PLoS One. 2026 Jun 30;21(6):e0351307. doi: 10.1371/journal.pone.0351307 (PMC13318022; doi:10.1371/journal.pone.0351307)
Supplement: S3 Table — (DOCX) [file pone.0351307.s003.docx]

**S3 Table. Sensitivity analysis of the association between categorized SHR and mortality after excluding critically ill patients.**

| **Variables** | **Model 1^a^** | |  | **Model 2^b^** | |
| --- | --- | --- | --- | --- | --- |
|  | **HR (95%CI)** | **P** |  | **HR (95%CI)** | **P** |
| 3 years |  |  |  |  |  |
| SHR < 1.08 | 1.00 (Reference) |  |  | 1.00 (Reference) |  |
| SHR ≥ 1.08 | 3.11 (1.92 – 5.04) | <.001 |  | 2.92 (1.76 – 4.86) | <.001 |
| 5 years |  |  |  |  |  |
| SHR < 1.08 | 1.00 (Reference) |  |  | 1.00 (Reference) |  |
| SHR ≥ 1.08 | 2.96 (1.85 – 4.73) | <.001 |  | 2.75 (1.68 – 4.50) | <.001 |

HR: Hazard Ratio, CI: Confidence Interval

^a^Model 1: Crude

^b^Model 2: Adjusted for gender, race, myocardial infarction, congestive heart failure, cerebrovascular disease, chronic pulmonary disease, renal disease, liver disease, metformin, insulin, age
